# Assessment of community pharmacists’ knowledge about drug-drug interactions in Jeddah, Saudi Arabia

**DOI:** 10.3389/fphar.2023.1209318

**Published:** 2023-06-01

**Authors:** Nasser M. Alorfi, Roaya S. Alqurashi, Alanood S. Algarni

**Affiliations:** Department of Pharmacology and Toxicology, College of Pharmacy, Umm Al-Qura University, Makkah, Saudi Arabia

**Keywords:** pharmacist, pharmacology, DDIs, counseling, quality

## Abstract

**Background:** Drug-drug interactions (DDIs) have the potential to result in severe adverse drug events and profoundly affect patient outcomes. The pivotal role community pharmacists assume in recognizing and effectively managing these interactions necessitates a comprehensive understanding and heightened awareness of their implications. Such knowledge and awareness among community pharmacists are fundamental for ensuring the delivery of safe and efficacious care to patients.

**Aim:** This study aimed to assess the knowledge of community pharmacists in Jeddah, Saudi Arabia, regarding drug-drug interactions (DDIs).

**Method:** A cross-sectional survey was administered to a cohort of 147 community pharmacists through the utilization of a self-administered questionnaire. The questionnaire encompassed a comprehensive range of 30 multiple-choice questions, encompassing various facets pertaining to drug-drug interactions (DDIs).

**Results:** A total of 147 community pharmacists working in Jeddah City, Saudi Arabia, completed the survey. The majority of them were male (89.1%, *n* = 131), and had bachelor’s degrees in pharmacy. Results showed that the lowest correct response of DDIs was between Theophylline/Omeprazole, while the highest was between amoxicillin and acetaminophen. Results revealed that among the 28 drug pairs, only six pairs were determined correctly by most participants. The study found that majority of the studied community pharmacist could not determine the correct answer on drug-drug interaction knowledge, as also seen with the measured below half mean DDIs knowledge of 38.22 ± 22.0 (min = 0, max = 89.29, median = 35.71).

**Conclusion:** The study highlights the need for ongoing training and education programs for community pharmacists in Saudi Arabia to enhance their knowledge and understanding of DDIs, ultimately leading to improved patient care and safety.

## Introduction

Drug-drug interactions (DDIs) occur when two or more drugs are administered simultaneously and interact with each other, resulting in altered pharmacological effects or toxicity. Its became a major concern in healthcare, particularly in the management of chronic diseases that require multiple medications (Obreli-Neto et al., 2012; Létinier et al., 2019; Hughes et al., 2023). DDIs can lead to adverse drug reactions, treatment failure, and even death (Bucşa et al., 2013; Chen et al., 2016; Ibrahim et al., 2021). Several factors can contribute to DDIs, including the pharmacokinetics and pharmacodynamics of the drugs involved (de Leon and Spina, 2018; Niu et al., 2019). Pharmacokinetic interactions involve alterations in the absorption, distribution, metabolism, and excretion of drugs, while pharmacodynamic interactions involve changes in the drug’s effects on the body. Some drugs may inhibit or induce the enzymes responsible for metabolizing other drugs, leading to altered drug concentrations and effects (Galetin et al., 2008; Almond et al., 2009). Other factors that can contribute to DDIs include genetic variations in drug metabolism, drug formulation, and patient-specific factors such as age, sex, and medical history (Akiyoshi et al., 2013; Tod et al., 2013; Malki and Pearson, 2019).

Community pharmacists play a crucial role in the prevention and management of DDIs by identifying potential interactions and providing appropriate advice to patients and other healthcare professionals (Becker et al., 2005; Chatsisvili et al., 2010; Hamadouk et al., 2022). The role of community pharmacists in improving patient outcomes through medication therapy management has been well established in the literature (Albabtain et al., 2021). One crucial aspect of medication therapy management is the identification and management of drug-drug interactions (DDIs), which can result in adverse drug events and decreased efficacy of medication (Juurlink, 2007). Community pharmacists are often the last point of contact between the patient and the healthcare system before medication use, and as such, play a critical role in identifying and managing potential DDIs (Vik et al., 2021). To enhance their ability to identify these interactions accurately and efficiently, community pharmacists increasingly rely on electronic databases specifically designed for checking DDIs. By harnessing the power of these databases, pharmacists can quickly and accurately identify potential interactions, ensuring patient safety and optimizing medication therapy.

In Saudi Arabia, community pharmacists are an integral part of the healthcare system, providing a range of services to the public, including dispensing medications, counseling patients, and monitoring drug therapy. However, there is limited research on the knowledge of community pharmacists about DDIs in Saudi Arabia. Assessing the knowledge of community pharmacists about DDIs is crucial for identifying areas of weakness and designing appropriate educational interventions to improve patient care and safety.

Several studies have been conducted in other countries to assess the knowledge of community pharmacists about DDIs, and they have reported variable levels of knowledge among pharmacists. However, there is a paucity of research on this topic in Saudi Arabia. Therefore, this study aimed to assess the knowledge of community pharmacists in Jeddah, Saudi Arabia, regarding DDIs. The findings of this study will provide valuable insights into the current state of knowledge of community pharmacists about DDIs in Jeddah and can inform the development of educational interventions to improve their knowledge and ultimately improve patient care and safety.

## Methods

### Sample size, sampling procedure and target population

According to the study, the sample size was determined using the total number of pharmacists working in the private sector in Jeddah city, which was 3,228 (Ministry of health, 2021). The sample size was calculated using the statistical program Openepi at a 90% confidence interval with a 5% margin of error, resulting in an estimated sample size of 250. Between November and December 2022, the questionnaire was electronically distributed to community pharmacists working in Jeddah via email and various social media platforms. Furthermore, pharmacy chain managers were contacted and asked to share the questionnaire with their pharmacists. The study was limited to community pharmacists working in Jeddah city, and those working in other parts of Saudi Arabia were not included in the study. To ensure the questionnaire’s quality, it was reviewed by two assistant professors of pharmacology. Following the review, a pilot study was conducted to assess face validity, involving fifteen community pharmacists who were not included in the final study.

### Study design and setting

For this study, a cross-sectional questionnaire was developed by the authors and administered to participants using a self-administered Google form. The questionnaire aimed to assess the knowledge level of community pharmacists regarding drug-drug interactions (DDIs) and was developed based on extensive research, studies, and clinical practices from around the globe. A list of 28 potential drug-drug interactions was asked of the pharmacists obtained from (Malone et al., 2004; Phansalkar et al., 2012; Al-Abdelmuhsin et al., 2021). The questionnaire employed in this study encompassed two distinct sections, encompassing a total of 34 multiple-choice items. The first section aimed to gather essential demographic data, encompassing variables such as age, gender, academic qualifications, years of professional experience, country of academic qualification, and the specific working region of each participant. This demographic information allowed for a comprehensive understanding of the characteristics and background of the participating pharmacists. The second section of the questionnaire was designed to assess the participants’ knowledge pertaining to drug-drug interactions (DDIs). This section involved a series of 28 commonly encountered drug pairs, meticulously selected based on their significant clinical impact and their frequency of occurrence within the literature. For each drug pair, participants were tasked with classifying the nature of the interaction into one of four distinct categories: “no interaction,” “contraindicated,” “with monitoring,” or “not sure.” These categories were chosen to enable a comprehensive evaluation of the participants’ understanding of the potential interactions between commonly used drugs. Recognizing the multitude of sources available for obtaining information about DDIs, participants were granted the flexibility to select multiple answers from the provided options. This approach allowed for a more accurate depiction of the participants’ reliance on various sources of drug information when assessing potential DDIs. The inclusion of these two sections within the questionnaire facilitated a comprehensive exploration of the participants’ demographic characteristics as well as their knowledge and understanding of DDIs. This enabled a thorough analysis of the relationship between demographic factors and DDI knowledge, shedding light on potential areas for improvement and targeted educational interventions within the community pharmacy setting.

### Statistical methodology and ethical consideration

The data was analyzed using IBM SPSS version 23 (IBM Corp., Armonk, N.Y., United States). Descriptive statistics were used to define the characteristics of the study variables, with categorical and nominal variables presented in counts and percentages, and continuous variables presented as mean and standard deviations. A scoring system was utilized to measure the level of knowledge of drug-drug interactions among community pharmacists in Jeddah. The study underwent review and approval by the institutional review board committee at the university (approval number (HAPO-02-K-012-2022-11-1303). Participation was voluntary, and all data collected were fully anonymized.

## Results

A total of 147 community pharmacists working in Jeddah City, Saudi Arabia completed the survey. The response rate was 58.8%. The data suggests that the male participants comprised a significantly higher percentage compared to the female participants. Specifically, males accounted for approximately 89% of the total participants, while females represented approximately 11%. The majority had bachelor’s degree in pharmacy (79.6%, n = 117), and had less than 10 years of experience in the field (51.0%, n = 75) (Table 1). The summary of 28 drug-drug interaction (DDI) knowledge of the pharmacists is indicated in Table 2.

**TABLE 1:** Socio-demographic characteristics of the studied community pharmacists (N = 147).

Demographics	Count	%
Total	147	100.0
*Age*		
21-25	5	3.4
26-30	40	27.2
31-36	58	39.5
Over 36	44	29.9
*Gender*		
Male	131	89.1
Female	16	10.9
*Pharmacy academic qualification*		
B.Pharm	117	79.6
PharmD	18	12.2
Postgraduate studies	12	8.2
*Years of experience*		
0–5 years	36	24.5
6–10 years	39	26.5
11–15 years	44	29.9
Above 15 years	28	19.0
*Country of Academic qualification*		
Local (Saudi Arabia)	44	29.9
Overseas	103	70.1

**TABLE 2:** Summary of 28 drug-drug interaction (DDI) knowledge of the studied pharmacists.

Drug–drug interaction pair		No interaction n (%)	Monitoring n (%)	Contraindication n (%)	Not sure n (%)
1	Digoxin/Erythromycin	8 (5.4%)	23 (15.6%)	90 (61.2%)	26 (17.7%)
2	Clarithromycin/Simvastatin	16 (10.9%)	27 (18.4%)	70 (47.6%)	34 (23.1%)
3	Phenytoin/Cimetidine	4 (2.7%)	31 (21.1%)	85 (57.8%)	27 (18.4%)
4	Itraconazole/Quinidine	4 (2.7%)	20 (13.6%)	79 (53.7%)	44 (29.9%)
5	Theophylline/Omeprazole	16 (10.9%)	44 (29.9%)	57 (38.8%)	30 (20.4%)
6	Sildenafil/Isosorbide mononitrate	6 (4.1%)	4 (2.7%)	120 (81.6%)	17 (11.6%)
7	Ibuprofen/Furosemide	23 (15.6%)	66 (44.9%)	23 (15.6%)	35 (23.8%)
8	Amoxicillin/Acetaminophen	113 (76.9%)	12 (8.2%)	3 (2.0%)	19 (12.9%)
9	Pimozide/Ketoconazole	16 (10.9%)	20 (13.6%)	53 (36.1%)	58 (39.5%)
10	Fluconazole/Phenytoin	11 (7.5%)	39 (26.5%)	56 (38.1%)	41 (27.9%)
11	Digoxin/Sildenafil	41 (27.9%)	24 (16.3%)	60 (40.8%)	22 (15.0%)
12	Alprazolam/Itraconazole	18 (12.2%)	15 (10.2%)	61 (41.5%)	53 (36.1%)
13	Fexofenadine HCL/Metoprolol	74 (50.3%)	27 (18.4%)	9 (6.1%)	37 (25.2%)
14	Amiodarone/Warfarin	7 (4.8%)	49 (33.3%)	54 (36.7%)	37 (25.2%)
15	Cyclosporine/Rifampicin	14 (9.5%)	37 (25.2%)	47 (32.0%)	49 (33.3%)
16	Raloxifene/Alendronate	39 (26.5%)	19 (12.9%)	31 (21.1%)	58 (39.5%)
17	Warfarin and sulfamethoxazole/Trimethoprim	8 (5.4%)	36 (24.5%)	60 (40.8%)	43 (29.3%)
18	Meloxicam/Gabapentin	77 (52.4%)	19 (12.9%)	14 (9.5%)	37 (25.2%)
19	Methotrexate/Probenecid	12 (8.2%)	31 (21.1%)	51 (34.7%)	53 (36.1%)
20	Phenytoin/Warfarin	6 (4.1%)	37 (25.2%)	66 (44.9%)	38 (25.9%)
21	Meperidine/Phenelzine	9 (6.1%)	19 (12.9%)	51 (34.7%)	68 (46.3%)
22	Rosuvastatin/Propranolol	67 (45.6%)	28 (19.0%)	11 (7.5%)	41 (27.9%)
23	Omeprazole/Clopidogrel	13 (8.8%)	31 (21.1%)	74 (50.3%)	29 (19.7%)
24	Amiodarone/Simvastatin	19 (12.9%)	36 (24.5%)	43 (29.3%)	49 (33.3%)
25	Diphenhydramine/Warfarin	54 (36.7%)	21 (14.3%)	28 (19.0%)	44 (29.9%)
26	Simvastatin/Itraconazole	18 (12.2%)	31 (21.1%)	55 (37.4%)	43 (29.3%)
27	Dopamine/Phenytoin	17 (11.6%)	35 (23.8%)	46 (31.3%)	49 (33.3%)
28	Acetaminophen/Celecoxib	84 (57.1%)	20 (13.6%)	12 (8.2%)	31 (21.1%)

Table 3 shows that among the 28 drug pairs, only six pairs were determined correctly by the majority of the participants, with 4 out of these 6 pairs having only <10% cutoff difference compared to its corresponding wrong answers. This suggests that still a majority of the studied community pharmacist could not determine the correct answer on DDI knowledge, as also seen with the measured below half mean DDIs knowledge of 38.22 ± 22.0 (min = 0, max = 89.29, median = 35.71). Twenty out of the 28 pairs exhibited statistically significant differences (*p* < 0.005).

**TABLE 3:** Correct *versus* incorrect answers of the studied pharmacists toward the 28 drug-drug interaction pair (DDI).

Drug–drug interaction pair			Incorrect n (%)	Correct n (%)	*p*-value
1	Digoxin/Erythromycin		124 (84.4%)	23 (15.6%)	<0.001^a^
2	Clarithromycin/Simvastatin		77 (52.4%)	70 (47.6%)	0.564
3	Phenytoin/Cimetidine		116 (78.9%)	31 (21.1%)	<0.001^a^
4	Itraconazole/Quinidine		68 (46.3%)	79 (53.7%)	0.364
5	Theophylline/Omeprazole		131 (89.1%)	16 (10.9%)	<0.001^a^
6	Sildenafil/Isosorbide mononitrate		27 (18.4%)	120 (81.6%)	<0.001^a^
7	Ibuprofen/Furosemide		81 (55.1%)	66 (44.9%)	0.216
8	Amoxicillin/Acetaminophen		34 (23.1%)	113 (76.9%)	<0.001^a^
9	Pimozide/Ketoconazole		94 (63.9%)	53 (36.1%)	0.001^a^
10	Fluconazole/Phenytoin		108 (73.5%)	39 (26.5%)	<0.001^a^
11	Digoxin/Sildenafil		106 (72.1%)	41 (27.9%)	<0.001^a^
12	Alprazolam/Itraconazole		86 (58.5%)	61 (41.5%)	0.039^a^
13	Fexofenadine HCL/Metoprolol		73 (49.7%)	74 (50.3%)	0.934
14	Amiodarone/Warfarin		93 (63.3%)	54 (36.7%)	0.001^a^
15	Cyclosporine/Rifampicin		110 (74.8%)	37 (25.2%)	<0.001^a^
16	Raloxifene/Alendronate		108 (73.5%)	39 (26.5%)	<0.001^a^
17	Warfarin and sulfamethoxazole/Trimethoprim		111 (75.5%)	36 (24.5%)	<0.001^a^
18	Meloxicam/Gabapentin		70 (47.6%)	77 (52.4%)	0.564
19	Methotrexate/Probenecid		96 (65.3%)	51 (34.7%)	<0.001^a^
20	Phenytoin/Warfarin		110 (74.8%)	37 (25.2%)	<0.001^a^
21	Meperidine/Phenelzine		96 (65.3%)	51 (34.7%)	<0.001^a^
22	Rosuvastatin/Propranolol		80 (54.4%)	67 (45.6%)	0.284
23	Omeprazole/Clopidogrel		73 (49.7%)	74 (50.3%)	0.934
24	Amiodarone/Simvastatin		111 (75.5%)	36 (24.5%)	<0.001^a^
25	Diphenhydramine/Warfarin		93 (63.3%)	54 (36.7%)	0.001^a^
26	Simvastatin/Itraconazole		92 (62.6%)	55 (37.4%)	0.002^a^
27	Dopamine/Phenytoin		112 (76.2%)	35 (23.8%)	<0.001^a^
28	Acetaminophen/Celecoxib		63 (42.9%)	84 (57.1%)	0.083
		**N**	**Min-Max**	**Mean ± SD**	**Median**
Knowledge on drug-drug interactions		147	0.00–89.29	38.22 ± 22.0	35.71

^a^ Significant using Chi-Square Test at 0.05 level.

Significant differences were found in the knowledge score of DDIs in terms of gender (*p* = 0.014) and years of experience (*p* = 0.007), as shown in Table 4. However, there were no significant differences were found between age groups, academic qualification, and country of academic qualification in the knowledge score of DDIs (*p* > 0.05). The result of test of between-subjects effects for factors such as gender and experiences are indicated in Table 5.

**TABLE 4:** Association between socio-demographic characteristic and knowledge on drug-drug interaction (DDI) of the studied community pharmacists.

Demographics	Total	Knowledge on drug-drug interactions	*p*-value
		Mean ± SD	
*Age*			
21-25	5	29.29 ± 17.9	0.258
26-30	40	41.96 ± 21.3	
31-36	58	34.61 ± 23.0	
Over 36	44	40.58 ± 21.5	
*Gender*			
Male	131	39.78 ± 22.0	0.014^a^
Female	16	25.45 ± 17.8	
*Pharmacy academic qualification*			
B.Pharm	117	40.14 ± 21.8	0.086
PharmD	18	28.37 ± 21.7	
Postgraduate studies	12	34.23 ± 22.3	
*Years of experience*			
0–5 years	36	34.82 ± 21.8^a^ ^,^ ^c^	0.007^b^ ^,^ ^c^
6–10 years	39	46.79 ± 22.1^b^	
11–15 years	44	31.25 ± 19.1^c^	
Above 15 years	28	41.58 ± 22.8^a^ ^,^ ^b^	
*Country of Academic qualification*			
Local (Saudi Arabia)	44	33.77 ± 21.3	0.110
Overseas	103	40.12 ± 22.2	

^a^ Significant using Independent *t*-test at <0.05 level.

^b^ Significant using One-Way ANOVA, Test at <0.05 level.

^c^ Post-Hoc Test = LSD.

* CAPITAL, letters indicates Post-Hoc multiple pairing summary indicator. Having the same letter means the same measure statistically.

**TABLE 5:** Tests of between-subjects effects.

Dependent variable: Knowledge on drug-drug interactions					
Source	Type III sum of squares	df	Mean square	F	*p*-value
Corrected Model	7,898.061^a^	4	1974.515	4.454	0.002
Intercept	57,023.426	1	57,023.426	128.622	<0.001
Gender	2,160.668	1	2,160.668	4.874	0.029
Years of experience	4,970.086	3	1656.695	3.737	0.013
Error	62,954.361	142	443.341		
Total	285,548.469	147			
Corrected Total	70,852.423	146			

Parameter Estimates					
Dependent variable: Knowledge on drug-drug interactions					
Parameter	B	S.E.	95% confidence interval		*p*-value
			Lower bound	Upper bound	
Intercept	28.345	7.196	14.119	42.570	<0.001^a^
Gender = Male	13.237	5.996	1.384	25.090	0.029^a^
Years of experience = 0–5 years	−2.716	5.613	−13.811	8.380	0.629
Years of experience = 6–10 years	5.892	5.225	−4.436	16.220	0.261
Years of experience = 11–15 years	−9.429	5.107	−19.524	0.666	0.067

^a^R Squared = 0.111 (Adjusted R Squared = 0.086).

^a^ Significant using General Linear Model at <0.05 level

## Discussion

This article about drug-drug interaction knowledge in Saudi Arabia sheds light on an important issue that affects patient safety and healthcare outcomes in the country. The findings of the study suggest that there is a significant lack of knowledge about drug-drug interactions among community pharmacists in Saudi Arabia, which could lead to adverse drug events and negative health outcomes for patients. One of the key points raised in the paper is the need for increased awareness and education about drug-drug interactions among healthcare professionals. This is an important first step towards improving patient safety and reducing the risk of adverse drug events. The study highlights the fact that many healthcare professionals in Saudi Arabia may not be adequately trained or equipped to identify and manage drug-drug interactions, which is a cause for concern. Among the pharmacists surveyed, it was observed that the majority, both in terms of percentage and actual numbers, were individuals from foreign countries. This indicates that the community pharmacy workforce in Saudi Arabia is largely comprised of professionals who originate from outside the country. Different studies have shown results within the demographics of community pharmacists practicing in the country. Our findings correlate with previous studies that revealed a noteworthy finding: a significant proportion of community pharmacists in Saudi Arabia are foreigners (Alkhuzaee et al., 2016; Rasheed et al., 2023). Moreover, the prevalence of foreign-educated community pharmacists in this study was generally high and also correlated with previous findings (Alaqeel and Abanmy, 2015; Hadi et al., 2016).

Several studies have examined the knowledge and awareness of community pharmacists regarding DDIs in different regions of the world. A study in Qatar, found that while most pharmacists recognized the potential for DDIs, there were gaps in their knowledge regarding specific interactions. For example, some pharmacists did not recognize the interaction between warfarin and nonsteroidal anti-inflammatory drugs (NSAIDs), which can increase the risk of bleeding. The study suggests that further training and continuing education may be necessary to improve pharmacists’ ability to manage DDIs (Abbas et al., 2022). Similarly, a study found that community pharmacists in the United States had a limited understanding of potential DDIs, particularly between nonprescription analgesics and prescription medications. The study suggests that pharmacists should receive more targeted education regarding these interactions, including the importance of patient counseling and medication review (Ylä-Rautio et al., 2020). In Greece, a study found that out of the 1,071 prescriptions analyzed, 663 (62%) had at least one potential DDI. Medical doctors have the responsibility of prescribing medications based on their clinical judgment and patient’s specific needs. They must consider factors such as the patient’s medical history, existing medications, allergies, and potential interactions when selecting and prescribing drugs. Effective communication, coordination, and sharing of information between medical doctors and pharmacists are essential to identify, prevent, and manage DDIs in patient care. The most common types of DDIs were pharmacokinetic interactions (60.4%), followed by pharmacodynamic interactions (34.2%) and pharmaceutical interactions (5.4%) (Chatsisvili et al., 2010). In this study, some of the factors were not significant. For example, there were no significant differences were found between age groups, academic qualification, and country of academic qualification in the knowledge score of DDIs (*p* > 0.05). This finding indicates that factors such as age, academic qualification, and the country where one received their academic qualification may not significantly influence the level of knowledge pharmacists possess regarding DDIs. It suggests that these factors may not be reliable predictors of knowledge in this specific domain. However, it is important to note that while the results did not find statistical significance, it does not necessarily mean that these factors have no influence at all. Other factors not examined in the study or interactions between multiple factors may still contribute to the knowledge scores.

Taken together, these studies suggest that community pharmacists’ knowledge and awareness of DDIs may be suboptimal in different regions of the world, despite their critical role in identifying and managing these interactions. The studies highlight the need for ongoing education and training programs, updated guidelines, and increased resources to support pharmacists in their efforts to provide safe and effective care to patients. Additionally, pharmacists may benefit from more targeted education regarding specific DDIs, including those between nonprescription and prescription medications, as well as the importance of patient counseling and medication review to identify and manage potential interactions. Particularly, improving pharmacists’ knowledge and awareness of DDIs can help to reduce the risk of adverse drug events and improve patient outcomes.

Overall, the article highlights an important issue that requires urgent attention in Saudi Arabia. Improving drug-drug interaction knowledge among community pharmacists is crucial for improving patient safety and healthcare outcomes in the country. By raising awareness of this issue and identifying potential solutions, your study makes an important contribution to the field of healthcare in Saudi Arabia and beyond.

## Conclusion and recommendations

The prevention and management of drug-drug interactions (DDIs) is of utmost importance in healthcare, and community pharmacists play a critical role in achieving this goal. The study has identified some recommendations to improve the knowledge and practice of community pharmacists regarding DDIs. Based on the study’s findings, it is recommended that continuing education and training programs should be developed for community pharmacists in Jeddah to improve their knowledge of DDIs. The Saudi Arabian Ministry of Health should develop guidelines and protocols for the management of DDIs in community pharmacies to ensure consistency in practice. Additionally, community pharmacies should have access to electronic databases that provide up-to-date information on DDIs to support their practice. Finally, future studies should be conducted to assess the impact of education and training programs on community pharmacists’ knowledge and practice regarding DDIs. In conclusion, ensuring that community pharmacists have adequate knowledge and skills is essential in preventing DDIs, and the above recommendations can help achieve this goal.

## Limitation

Firstly, the study’s sample size may limit the generalizability of the findings. If the sample size is small or not representative of the entire population of community pharmacists in Jeddah, the results may not accurately reflect the overall knowledge level of pharmacists in the region. Additionally, the study’s reliance on self-reported data from community pharmacists introduces the possibility of response bias. Participants may overestimate their knowledge to present themselves in a more favorable light or may underreport their knowledge about DDIs due to various reasons, such as social desirability bias. Furthermore, the study focuses solely on assessing community pharmacists’ knowledge without considering other factors that may influence their ability to apply that knowledge in practice, such as time constraints, workload, or access to resources.

## Data Availability

The raw data supporting the conclusions of this article will be made available by the authors, without undue reservation.
